# *Restless Legs Syndrome* and Impulsive Decision‐Making: Impact of Symptom Severity, Chronotype and Interoception

**DOI:** 10.1002/mdc3.70040

**Published:** 2025-03-18

**Authors:** Giacomo Carollo, Riccardo Quinci, Gloria Pompea Mingolla, Angela Sandri, Michele Tinazzi, Elena Antelmi

**Affiliations:** ^1^ DIMI, Department of Engineering and Medicine of Innovation University of Verona Verona Italy; ^2^ Department of Medicine and Surgery University of Verona Verona Italy; ^3^ Department of Neurosciences, Biomedicine and Movement Sciences University of Verona Verona Italy; ^4^ Neurology Unit, Parkinson Disease and Movement Disorders Division University of Verona Verona Italy

**Keywords:** restless legs syndrome, interoception, alexithymia, chronotype, decision making

## Abstract

**Background:**

Restless Legs Syndrome (RLS) is a neurological disorder reported to be associated with impulsivity and impairments in interoception and emotional regulation. However, limited research has explored the combined influence of RLS severity, psychological factors, and chronotype on impulsive decision‐making and risk‐taking behavior in RLS patients.

**Objectives:**

To assess impulsive‐decision making and its modulation by RLS severity, chronotype, and psychological factors (including interoception, anxiety, alexithymia, and sleep quality) in RLS patients compared to healthy controls (HC).

**Methods:**

A case–control study was conducted with 20 RLS patients and 20 age‐ and sex‐matched HC. Participants completed a series of questionnaires measuring interoception, anxiety, alexithymia, sleep quality, and chronotype, followed by a temporal discounting task to assess impulsivity. Statistical analyses included Mann–Whitney U tests, Spearman's rank correlations, and multiple regression analysis.

**Results:**

No significant differences in impulsive decision‐making were observed between groups. RLS patients exhibited higher levels of depression and alexithymia, along with lower scores on interoceptive awareness compared to HC. However, within the RLS group, greater symptoms’ severity, poorer sleep quality, and higher anxiety were positively correlated with increased impulsivity. Regression analysis showed that the MAIA‐2‐Not Distracting sub‐scale was a significant predictor of impulsivity in RLS patients.

**Conclusions:**

Interoceptive deficits, particularly difficulties in ignoring bodily sensations, play a central role in impulsive decision‐making in RLS patients. These findings highlight the importance of targeting interoception, emotional regulation, and sleep quality in therapeutic interventions for RLS. Further research with larger samples is needed to confirm these relationships.

Restless Legs Syndrome (RLS) is a prevalent neurological disorder characterized by an uncontrollable urge to move the legs, typically accompanied by uncomfortable sensations and more prevalent in women.^1^, ^2^ These symptoms predominantly worsen during rest and towards the evening, impacting sleep quality and overall well‐being.^3^ The pathophysiology of RLS is believed to involve central nervous system pathways including dopaminergic dysfunction and defect in brain iron metabolism.^1^, ^2^ In recent years, the psychological dimensions of RLS have gained increased attention, particularly in relation to interoception—defined as the awareness of internal bodily states— as well as emotional regulation and decision‐making processes.^4^, ^5^, ^6^, ^7^, ^8^ Impairments in these areas have been frequently reported among individuals with RLS and are believed to be exacerbated by the presence of alexithymia, a condition that involves difficulties in recognizing and expressing emotions.^9^, ^10^ Alexithymia has been shown to correlate with interoceptive deficits and has a notable impact on decision‐making abilities in both healthy individuals and clinical populations.^11^, ^12^, ^13^ In particular, RLS patients, with or without impulse control disorders, exhibit deficits in decision making compared to healthy controls.^14^, ^15^

Despite these insights, few studies have specifically examined how these cognitive and emotional factors are modulated by the severity of RLS symptoms. Moreover, the role of chronotype, or an individual's circadian preference,^16^ in influencing psychological and sensorimotor dimensions in RLS has yet to be fully explored, even though RLS symptoms are closely linked to circadian rhythms. Given the circadian nature of RLS symptoms,^1^ we hypothesized that chronotype might modulate psychological and sensorimotor dimensions, influencing decision‐making and impulsivity.

This study aims to address these gaps by investigating risk‐taking behaviors, particularly in relation to decision‐making and impulsivity, in RLS patients. Additionally, we will examine how these behaviors are influenced by RLS symptom severity, psychological factors (including alexithymia and interoception), and chronotype. Our findings could help develop more targeted psychological interventions to improve decision‐making and emotional regulation in individuals with RLS, ultimately enhancing their quality of life.

## Methods

### Participants

In this case–control study, we compared individuals diagnosed with idiopathic Restless Legs Syndrome (RLS), according to the latest international criteria,^17^ to healthy controls (HC) matched for age and sex. Subjects were consecutively recruited from the sleep disorder outpatient clinic of Borgo Roma Verona University Hospital, Neurology Unit. Inclusion criteria were: age over 18 years old, possessing a valid and functional email address, and having adequate digital skills for accessing email, following external links, and navigating web pages. Exclusion criteria included current augmentation due to RLS pharmacotherapy, major psychiatric or comorbid neurological disorders and the use of medications known to affect decision‐making capabilities, such as antipsychotics, opioids, and CNS depressants like benzodiazepines and barbiturates.^1^, ^2^ These drugs were excluded to minimize confounding effects on cognitive performance. Informed consent was obtained from all participants, and the study was approved by the local institutional review board (Prog. 3049CESC). Initially, 36 RLS patients were approached; however, 11 patients were excluded due to comorbid neurological or psychiatric conditions, and five were excluded for lacking access to email or internet skills. Thus, 20 patients (15 females, mean age 57.15 years ±10.58) with a primary clinical diagnosis of RLS and 20 HC (12 females, mean age 53.70 years ±11.13) were included. RLS symptom severity was measured using the International RLS Severity Scale (IRLSSS).^18^ Eligibility criteria were initially assessed via self‐reported data but subsequently verified through clinical review by investigators to ensure accuracy and adherence to inclusion criteria.

#### Washout Period

In the RLS group, six participants treated with dopamine agonists (eg, pramipexole) underwent a 2‐day washout period to minimize medication effects on decision‐making.^1^, ^19^ Four participants on alpha‐2 delta ligands (eg, gabapentin) and three on benzodiazepines did not require a washout. Seven participants reported no medication use. A 2‐day washout was selected to balance minimizing residual effects with maintaining patient safety, as longer washouts were impractical for this study.

No cases of augmentation were identified in our cohort, based on comprehensive clinical interviews evaluation present and past symptoms of augmentation.

### Procedure

Participants recruited at the Principal Investigator's outpatient sleep clinic were each sent two separate, unique LimeSurvey email links, between 3:00 and 5:00 p.m., with telephone support available if necessary. The first link was used to conduct an online screening, where eligibility was assessed based on demographic and health criteria. Those who passed the screening were provided with a comprehensive explanation of the study's objectives and procedures before giving their digital consent. Upon consent, participants received a second, unique email link to complete the main survey, where participants completed a series of psychological questionnaires, and an online behavioral task aimed at evaluating impulsive decision‐making. Augmentation was assessed through detailed clinical interviews and patient history, but no cases were identified among the participants.

Healthy controls (HC) were recruited through internal university advertisements and invitation letters, which participants were encouraged to share with friends or family members to invite them to participate. All participants underwent screening with clinical interviews according to international criteria^17^ to exclude RLS symptoms and any family history of RLS.

### Intertemporal Decision Making Task

Participants completed a Temporal Discounting Task to evaluate impulsive decision‐making involving three binary choice questions.^20^ Each question presented participants with a hypothetical decision between a smaller, immediate reward and a larger, delayed reward. For example, participants were asked: “Would you prefer €1000 now or €1100 in one year?” The immediate reward was fixed at €1000, while the delayed reward varied between €1100 and €1500, with a constant delay of 1 year. This version of the task, adapted for Italian participants, demonstrated adequate internal consistency with a Cronbach's alpha of 0.69 (see supplementary material in Data S1).

### Questionnaire Assessment

We administered the Hospital Anxiety and Depression Scale (HADS)^21^ to assess anxiety and depression, the Toronto Alexithymia Scale (TAS‐20)^22^, ^23^ to evaluate the ability to perceive and recognize emotions and the Multidimensional Assessment of Interoceptive Awareness (MAIA‐2)^24^ to evaluate different aspects of interoception. The Insomnia Severity Index ISI^25^, ^26^ was used to evaluate insomnia severity, while chronotype was assessed using the Reduced Morningness‐Eveningness Questionnaire (R‐MEQ).^27^ Finally, night eating behaviors were assessed through one initial question in the survey (ie “Do you ever eat at night?”) and participants who answered positively were directed to the compilation of the Italian Night Eating Questionnaire (I‐NEQ).^28^

### Statistical Analysis

Descriptive statistics, normality tests (Shapiro–Wilk), and homogeneity tests (Levene's test) were applied to demographic and clinical variables. Since data was not normally distributed, non‐parametric analyses were performed. Comparisons between groups were made using Fisher's exact tests and Mann–Whitney U tests. To control for multiple comparisons in the comparative analysis of MAIA‐2 subscales, the False Discovery Rate (FDR) correction was applied using the Benjamini‐Hochberg method. This approach was chosen as it is less conservative than the Bonferroni correction, thereby reducing the risk of type II errors while maintaining adequate control over type I errors.

To evaluate differences at the intertemporal decision‐making task, an individual discount rate was calculated for each participant using a hyperbolic discount model.^20^ This model estimates the subjective value of a delayed reward (V) based on the formula:

$$V=\frac{A}{1+\mathrm{kD}}$$

where A is the amount of the delayed reward, D is the delay (in this case, 1 year), and k is the individual's discount rate. Larger values of k indicate a stronger preference for immediate rewards, reflecting greater impulsivity. The participants’ responses across the three questions were used to estimate their individual discount rate, with higher rates reflecting a greater tendency to favor smaller, immediate rewards over larger, delayed ones.

Spearman's rank correlations were employed to explore associations in RLS patients between the calculated discount rate and psychological and clinical measures, such as RLS severity (IRLSSS), insomnia severity (ISI), interoceptive awareness (MAIA‐2), anxiety and depression symptoms (HADS), alexithymia (TAS‐20). Additionally, a multiple linear regression analysis was conducted to investigate predictors of impulsive decision‐making (discount rate) within the RLS group. The regression model included relevant variables of interest which emerged from correlations with discount rate, specifically RLS symptom severity, insomnia severity, anxiety and interoceptive awareness (MAIA‐2‐Not Distracting) as covariates to assess their contribution to impulsivity. The model's significance was evaluated using an F‐test, and collinearity diagnostics (Variance Inflation Factor) were performed to assess multicollinearity between predictors.

Analyses were performed using Jamovi (version 2.5) and the Python programming language with the “SciPy” library for non‐parametric tests, and the “statsmodels” library for regression analyses and multiple comparisons correction.

## Results

### Demographic Characteristics

Clinical and demographic characteristics are shown in Table 1. According to IRLSSS cutoffs,^18^ patients presented severe RLS symptoms (Median 30.0 [IQR = 11.80]). As expected, the two groups did not differ for age nor gender (both *p* > 0.05). Regarding years of education, patients significantly had less years (U = 112.5, *p* = 0.013). Finally, there were no significant differences in the number of smokers between the groups, (odds ratio = 2.15, *p* > 0.05). Seven RLS participants versus four HC participants reported night eating behaviors, with no significant difference between groups (*p* > 0.05; see Table 1).

**TABLE 1:** Participants' clinical and demographic characteristics

	RLS (*N* = 20)	HC (*N* = 20)	*p*‐values
Age, years (*±* SD)^a^	57.15 (*±* 10.58)	53.70 (*±* 11.13)	0.321
Women, no. (%)^b^	15 (75.0)	12 (60.0)	0.501
Years of education, median (IQR)^a^	13.0 (5.75)	14.5 (5.00)	0.013*
Smokers, no. (%)^b^	5 (25.0)	1 (5.0)	0.182
Night eaters, no. (%)^b^	7 (35.0)	4 (20.0)	0.480
Symptoms’ duration—years, median (IQR)	16.50 (22.25)	–	–

Abbreviations: RLS, Restless Legs Syndrome; HC, Healthy Controls; SD, Standard Deviation; IQR, Interquartile Range.

^a^ Mann–Whitney U test.

^b^ Fisher's exact test.

### Intertemporal Decision‐Making Task

**Figure 1:**
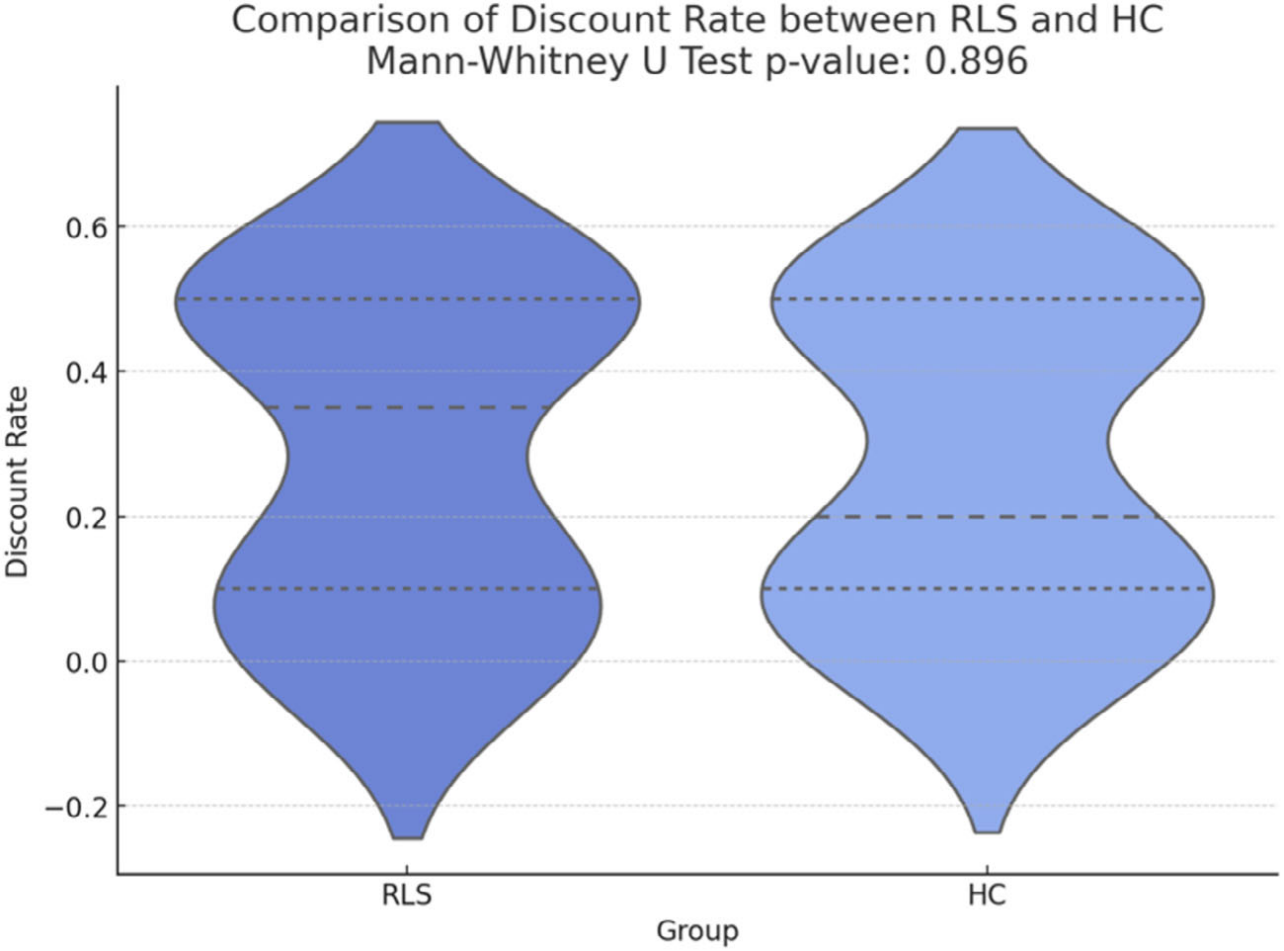
Violin plot distribution of the discount rate in RLS and HC groups. No significant difference was found between the groups (U = 195.0, *p* = 0.896). Dashed lines represent median values, dotted lines represent the 25th and 75th percentiles.

To evaluate differences in intertemporal decision making between RLS and HC, we compared the discount rates across both groups and found no significant differences (U = 195.0, *p* > 0.05; Fig. 1).

### Psychological Measures

Questionnaires scores can be seen in Table 2. RLS participants had significantly higher scores on measures of depression (HADS‐Depression) and alexithymia (TAS‐20; both *p* < 0.01), but not for anxiety (*p* > 0.05).

**TABLE 2:** Psychological Questionnaires Results

	RLS (*N* = 20)	HC (*N* = 20)	*p*‐values
TAS‐20, median (IQR)^a^	58.0 (12.75)	42.0 (17.25)	0.0087*
HADS Anxiety, median (IQR)^a^	8.0 (3.75)	7.0 (4.25)	0.0681
HADS Depression, median (IQR)^a^	8.0 (5.25)	3.0 (3.00)	0.0025*
ISI, median (IQR)^a^	14.5 (10.25)	3.5 (4.00)	0.0001*
R‐MEQ, median (IQR)^a^	15.0 (5.50)	15.5 (6.00)	0.4134

Abbreviations: RLS, Restless Legs Syndrome; HC, Healthy Controls; IQR, Interquartile Range; TAS‐20, Toronto Alexithymia Scale – 20 items; HADS, Hospital Anxiety and Depression Scale; ISI, Insomnia Severity Index; R‐MEQ, Reduced Morningness‐Eveningness Questionnaire.

^a^ Mann–Whitney U test.

**Figure 2:**
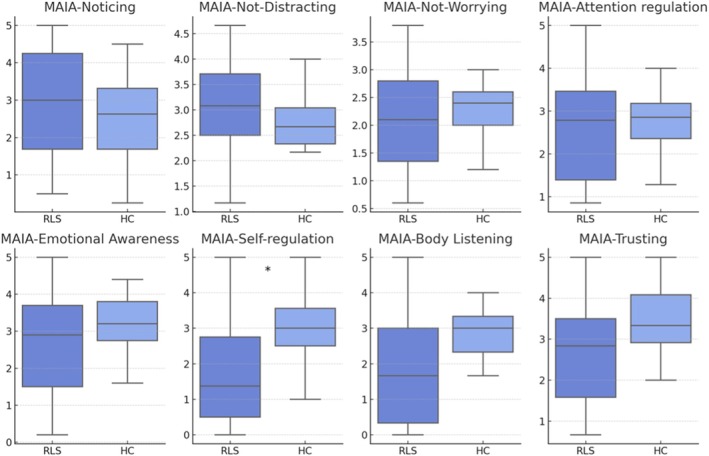
Boxplot of MAIA‐2 components for RLS patients and controls. Boxplot of data for each component of MAIA‐2 questionnaire for RLS patients (blue) and HC (light blue). Bold lines represent median values. * = *p* < 0.05.

For the MAIA‐2 subscales (Fig. 2), the comparison between groups showed a significant difference after correction in the “MAIA‐2‐Self‐regulation” subscale, indicating a significantly lower self‐regulation ability in the RLS group (median RLS group = 1.38 [IQR = 2.25], median HC group = 3 [IQR = 1.06], corrected *p* = 0.028). The remaining subscales showed no significant differences.

The analysis on chronotype (R‐MEQ scores) between groups showed no significant difference (U = 170, *p* > 0.05), while regarding ISI scores a significant difference was revealed (U = 45, *p* < 0.001), suggesting that RLS patients have significantly higher insomnia severity compared to HC (Table 2).

### Correlation Analyses

**Figure 3:**
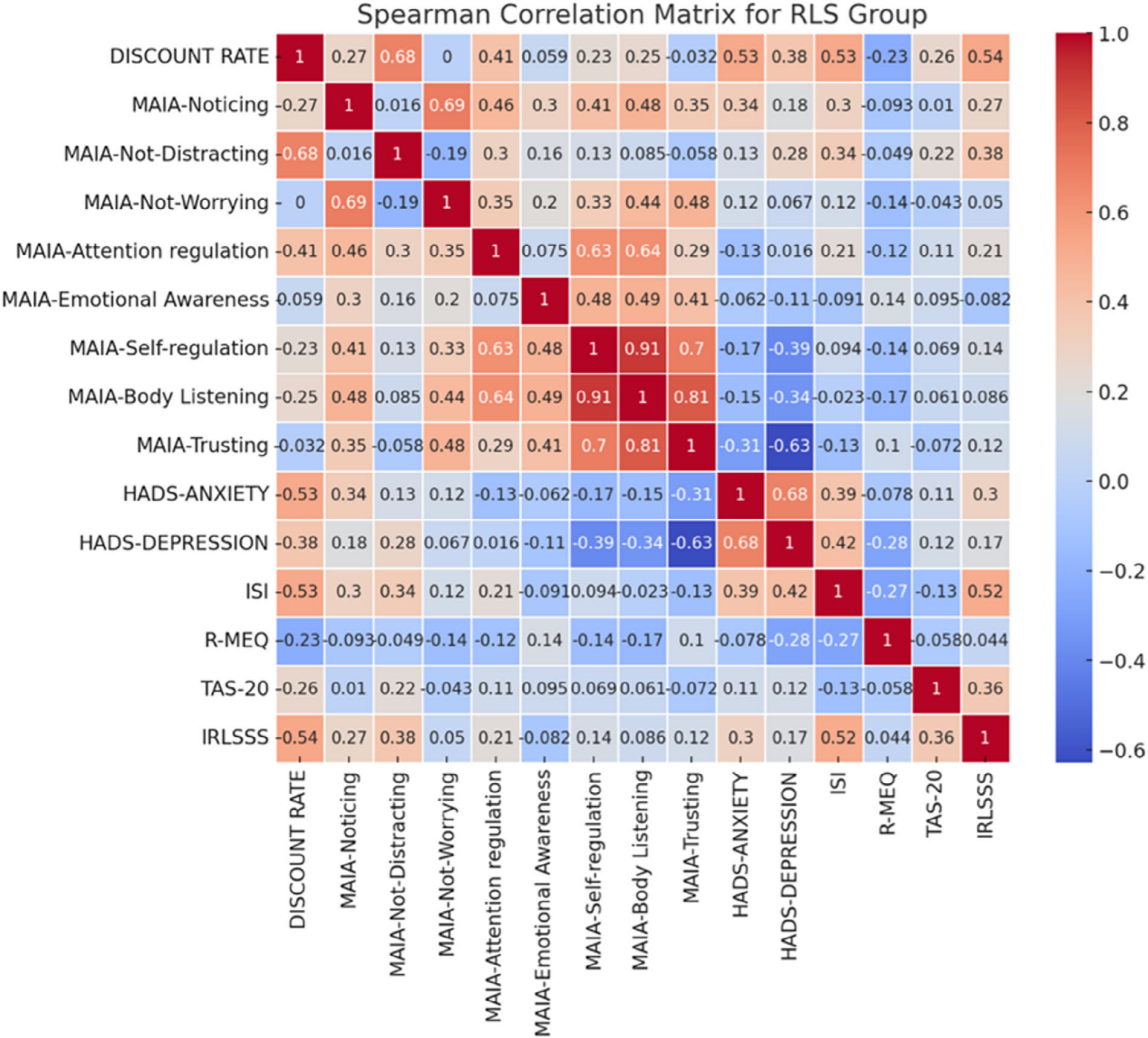
Spearman's correlation matrix for RLS group. Correlations between clinical data, questionnaires scores and performance at the intertemporal decision‐making task. Numbers reported represent the Pearson's coefficients for each correlation. Darker colors mean stronger and significant positive (red) or negative (blue) correlations.

**Figure 4:**
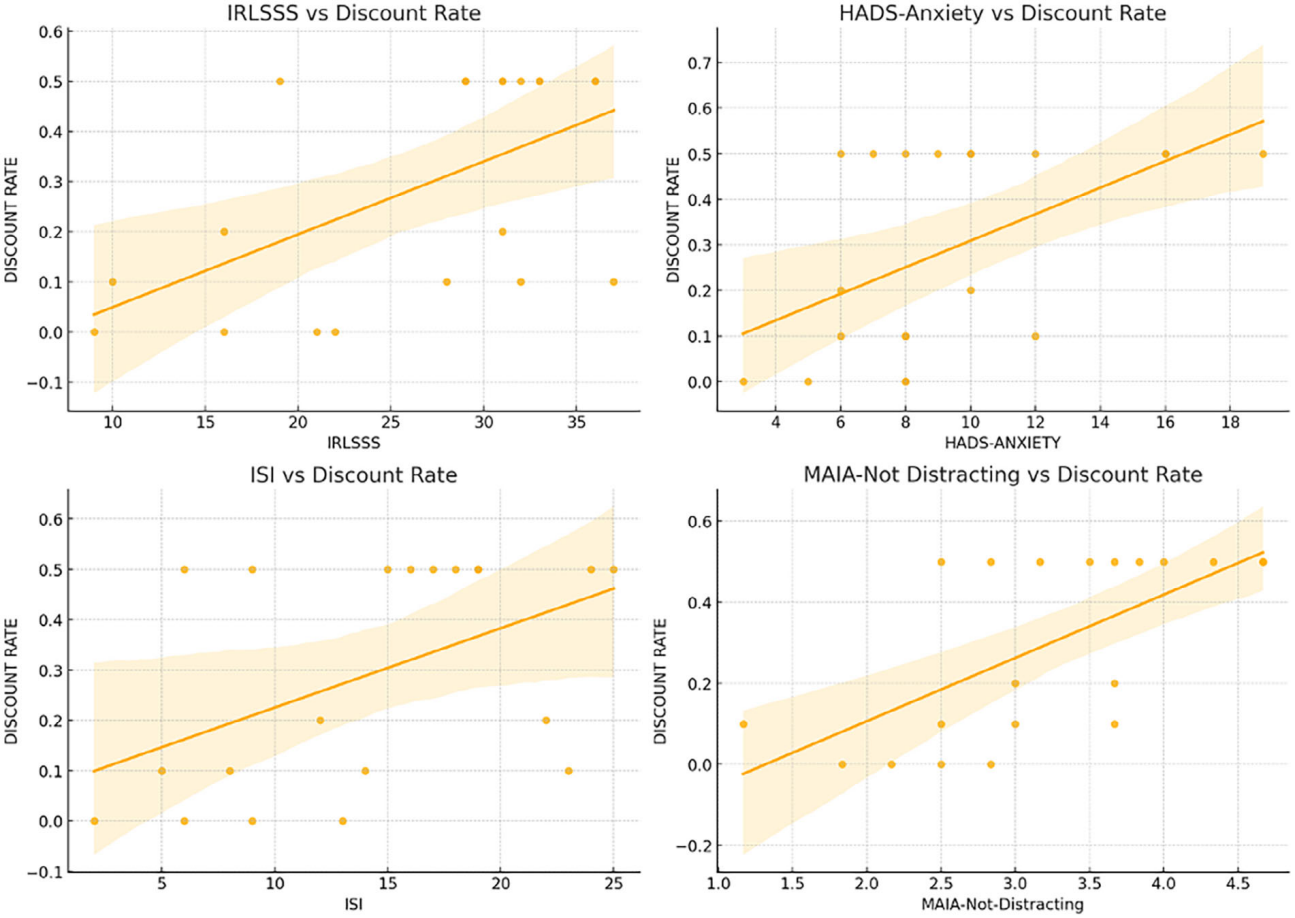
Significant Spearman's correlations between discount rate and IRLSSS (upper left, ρ = 0.535, *p* = 0.015), HADS‐Anxiety (upper right, ρ = 0.529, *p* = 0.016), ISI (lower left, ρ = 0.528, *p* = 0.017), and MAIA‐2‐Not‐Distracting (lower right, ρ = 0.677, *p* = 0.001).

For the within‐group analyses of the RLS population, Spearman's rank correlation revealed several significant associations between the discount rate and clinical/psychological measures (see Figs. 3 and 4). Significant positive correlations were observed between the discount rate and RLS symptom severity (IRLSSS; ρ = 0.535, *p* = 0.015), sleep quality (ISI score; ρ = 0.528, *p* = 0.017), anxiety (HADS‐Anxiety score; ρ = 0.529, *p* = 0.016), and interoceptive awareness (MAIA‐2‐Not Distracting subscale; ρ = 0.677, *p* = 0.001). Other variables did not show significant correlations with the discount rate.

No significant correlation was found between chronotype (R‐MEQ) and the discount rate, nor between chronotype and RLS symptom severity (both, *p* > 0.05).

### Regression Analysis

A multiple linear regression analysis was conducted to explore the predictive role of RLS symptom severity (IRLSSS), interoceptive awareness (MAIA‐2‐Not Distracting), sleep quality (ISI), and anxiety (HADS‐Anxiety) on impulsive decision‐making (discount rate) within the RLS group. The overall model was statistically significant (*F*[4,15] = 7.03, *p* = 0.002), explaining 65.2% of the variance in discount rate (*R* ^2^ = 0.652), with an adjusted R^2^ of 0.559.

Among the predictors, MAIA‐2‐Not Distracting emerged as a significant positive predictor of the discount rate (β = 0.114, *p* = 0.011), indicating that higher tendencies to not distract from bodily sensations were associated with increased impulsivity in decision‐making. None of the other predictors showed significant associations with the discount rate.

Collinearity statistics (VIF) indicated no multicollinearity issues among the predictors, with all VIF values below 2.0, confirming that the variables included in the model were independent of each other.

The model was re‐ran adjusting for education years and results were confirmed (data available from the authors).

## Discussion

These findings provide a deeper understanding of the psychological and sensorimotor dimensions of Restless Legs Syndrome (RLS). Specifically, in our sample, decision‐making was largely preserved, as indicated by comparable scores at the intertemporal decision‐making task between RLS patients and healthy controls (HC). However, among RLS patients, worse symptom severity (IRLSSS), interoceptive deficits (MAIA‐2‐Not Distracting), anxiety (HADS‐A) and poorer sleep quality (ISI) were significantly correlated with higher discount rates in the temporal discounting task, suggesting high impulsivity. Eventually, interoceptive deficits seem to be involved in impulsive decision‐making.

The fact that we did not find significant differences in decision‐making between RLS patients and controls contrasts with prior studies that highlighted impairments in RLS patients. For instance, Bayard et al^14^ observed that RLS patients performed worse on the Iowa Gambling Task (IGT), though no differences were found on the Game of Dice Task (GDT), while Heim et al^15^ showed that RLS patients, regardless of dopamine‐agonist treatment, made more irrational decisions and gathered less information in the Beads Task, but performed similarly to controls in the Pixel Task. These findings suggest that decision‐making difficulties in RLS may be more pronounced in tasks involving ambiguity, rather than those requiring immediate judgments or calculated risks. Furthermore, previous studies^29^, ^30^ documented that RLS patients experiencing augmentation (symptom worsening due to dopaminergic treatment) were more likely to exhibit impulse control disorders, indicating that impulsive behaviors in RLS may be more closely tied to augmentation rather than the condition itself. A further possible explanation for the lack of significant findings in our study could be the relatively small sample size (20 RLS patients and 20 controls) or the specific decision‐making task used, which may not have been sensitive enough to detect subtle differences in behavior.

The results of the linear regression provide further insights, suggesting that while symptom severity, sleep quality and anxiety are correlated with impulsivity, MAIA‐2‐Not Distracting, a measure of interoceptive awareness, was the only significant predictor of discount rate. This suggests that RLS patients who are more focused on bodily sensations, such as discomfort or pain, are more likely to make impulsive decisions, preferring immediate rewards over delayed ones. This finding highlights the role of interoceptive awareness in impulsive decision‐making, aligning with the concept of “hot” impulsivity, where interoceptive sensibility influences affect‐driven preferences for immediate gratification in the presence of anticipatory cues.^31^

The significant positive correlation between the discount rate and the MAIA‐2‐Not Distracting subscale supports the idea that RLS patients who are more prone to focus on sensations of pain or discomfort are also more likely to make impulsive decisions. Interoceptive deficits, such as lower scores in self‐regulation, body listening, and trust in one's body, may exacerbate symptoms and overall well‐being in RLS patients, as previously reported.^8^

Poor sleep quality and greater symptom severity were significantly associated with increased impulsivity in the RLS group. Sleep deprivation is known to impair cognitive functions such as attention, memory, and executive functions, which are essential for inhibitory control and decision‐making.^32^, ^33^ The overlap between RLS and chronic insomnia complicates diagnosis and may exacerbate the cognitive deficits observed in these patients.^34^ However, despite this strong correlation, sleep quality did not emerge as a significant predictor in the regression model, suggesting that interoceptive awareness may play a more central role in driving impulsive behaviors in RLS patients.

We observed that impulsivity in RLS patients is intricately linked to symptom severity, interoception, and emotional regulation. Previous findings^30^ also highlighted the role of augmentation in exacerbating impulsivity and alexithymia. In fact, RLS patients with augmentation exhibit significant impairments in emotion recognition, particularly for negative emotions, and increased alexithymia scores. These traits were attributed to dopaminergic dysregulation and neuroplastic changes, which could similarly influence impulsive behaviors observed in our study cohort.^35^, ^36^, ^37^, ^38^, ^39^ Moreover, Cavallaro et al^37^ found that increased dopamine D2 receptor (D2R) levels in nucleus accumbens cholinergic interneurons (CINs) led to more impulsive choices in delay discounting tasks, suggesting that RLS patients with more severe symptoms and greater dopaminergic dysfunction may exhibit heightened impulsivity, independent of sleep quality.

This study also confirms previous research showing impairment in interoception, emotional regulation, and higher levels of alexithymia and depression in RLS patients compared to HC.^8^, ^12^ Interestingly, chronotype did not differ significantly between RLS patients and controls, although circadian rhythmicity plays a critical role in RLS clinical features.^1^

Furthermore, the higher levels of alexithymia and depression observed in RLS patients compared to controls underscore the importance of psychological factors in the manifestation and management of RLS.^39^ Alexithymia, which has been linked to poorer decision‐making even in non‐clinical populations,^12^ may contribute to the emotional and interoceptive deficits in RLS, as alexithymic patients often struggle with both emotional and non‐affective interoceptive signals such as hunger and fatigue.^40^

This study also explored the role of chronotype in RLS, finding no significant differences between RLS patients and controls, despite the circadian nature of RLS symptoms.^1^ While the experimental tasks were conducted in a single time frame (between 3:00 and 5:00 p.m.), assuming stability of economic decision‐making across circadian phases,^41^ future studies should explore how time‐of‐day variations may impact decision‐making abilities in RLS patients.

Despite its strengths in assessing multiple psychological factors, this study has limitations. The small sample size limits the generalizability of the findings and prevents more detailed subgroup analyses. Moreover, the reliance on self‐report questionnaires introduces potential response biases,^42^ and incorporating actigraphy or polysomnography in future studies would provide objective measures of sleep quality, complementing subjective self‐reports and reducing bias.

Another limitation is the educational disparity between the patient and control groups, with HC having a significantly higher level of education. Although education is generally associated with better decision‐making^43^, ^44^, ^45^ it did not appear to influence the outcomes in this study, as no significant differences in decision‐making were found between the groups. Lastly, the cross‐sectional design of the study limits the ability to establish causal relationships between the variables.

The 2‐day washout period represents a limitation, as residual medication effects could influence decision‐making outcomes. Future studies might benefit from extended washout durations, although this could pose logistical challenges.

Subgroup analyses should be interpreted cautiously due to the small sample size in the RLS group. While our findings provide preliminary insights, they require validation in larger, more diverse samples.

Given these preliminary results, future research exploring interventions aimed at improving interoceptive awareness, emotional regulation, and sleep quality could lead to targeted therapies that significantly improve the quality of life for RLS patients and reduce symptom severity.

## Author Roles

(1) Research project: A. Conception, B. Organization, C. Execution; (2) Statistical Analysis: A. Design, B. Execution, C. Review and Critique; (3) Manuscript Preparation: A. Writing of the First Draft, B. Review and Critique.

G.C.: 1B, 1C, 2A, 2B, 3A

R.Q.: 1B, 1C

G.P.M.: 2C, 3B

A.S.: 2C, 3B

M.T.: 2C, 3B

E.A.: 1A, 1B, 1C, 2C, 3B

## Disclosures

**Ethical Compliance Statement:** The local institutional review board of Verona University approved the protocol (Prog. 3049CESC), and all patients provided written informed consent. All authors confirm that they have read the Journal's position on issues involved in ethical publication and affirm that this work is consistent with those guidelines.

**Funding Sources and Conflicts of Interest:** No specific funding was received for this work, and the authors declare that there are no conflicts of interest relevant to this work.

**Financial Disclosures for the Previous 12 Months:** All authors declare that there are no additional disclosures to report.

## Supporting information


**Data S1.** The supplementary file presents a pilot study validating the Italian version of the Intertemporal Decision Making Task, originally described by Boyle et al, 2013 (20). The study aimed to assess the internal consistency and reliability of the translated questionnaire for future research on intertemporal decision‐making in Italian populations. A sample of 30 healthy participants completed the task, which involved three binary‐choice questions comparing immediate and delayed rewards. Statistical analyses, including Cronbach's alpha, confirmed acceptable internal consistency (α = 0.693), suggesting that the Italian version maintains the psychometric properties of the original instrument. The findings support the usability of this tool in Italian studies, with further validation recommended in larger samples.

## Data Availability

The data that support the findings of this study are available on request from the corresponding author. The data are not publicly available due to privacy or ethical restrictions.
