# Supplementary material for: Restless Legs Syndrome and Impulsive Decision‐Making: Impact of Symptom Severity, Chronotype and Interoception
Source: Mov Disord Clin Pract. 2025 Mar 18;12(8):1066–74. doi: 10.1002/mdc3.70040 (PMC12371472; doi:10.1002/mdc3.70040)
Supplement: Supplementary file 1 — Data S1. The supplementary file presents a pilot study validating the Italian version of the Intertemporal Decision Making Task, originally described by Boyle et al, 2013 (20). The study aimed to assess the internal consistency and reliability of the translated questionnaire for future research on intertemporal decision‐making in Italian populations. A sample of 30 healthy participants completed the task, which involved three binary‐choice questions comparing immediate and delayed rewards. Statistical analyses, including Cronbach's alpha, confirmed acceptable internal consistency (α = 0.693), suggesting that the Italian version maintains the psychometric properties of the original instrument. The findings support the usability of this tool in Italian studies, with further validation recommended in larger samples. [file MDC3-12-1066-s001.docx]

**SUPPLEMENTARY FILE**

*Restless Legs Syndrome* and impulsive decision-making: Impact of Symptom Severity, Chronotype and Interoception

**Introduction**

This document describes a pilot study conducted to validate the Italian version of the Intertemporal Decision Making Task originally described by Boyle et al. [1]. The primary objective was to assess the internal consistency and reliability of the literal translation of the original items, aiming to use it in future studies on intertemporal decision-making among Italian populations.

**Methods**

**Participants**

The study involved 30 healthy participants recruited through an anonymous internal survey at the University of Verona. The sample comprised 16 males (53.33%) and 14 females (46.67%), with an age range that produced a mean of 41.5 years, a standard deviation of 8.95 years, a median of 44 years, and an interquartile range (IQR) of 13 years. All participants provided informed consent prior to participation.

**Instrument**

The Intertemporal Decision Making Task employed in Boyle et al. [10] consists of three binary-choice questions presenting hypothetical decisions between a smaller immediate reward and a larger delayed reward. In the original task, participants were asked “ Which do you prefer, that you get $1000 in cash right now or [delayed reward] in a year?” The immediate reward was fixed at €1000, while the delayed reward varied between $1100, $1200 and $1500, with a constant delay of one year. In the Italian version employed for this study, the same question was translated in “Preferirebbe ricevere €1000 in contanti adesso oppure [delayed reward] tra un anno?”.

**Procedure**

The literal translation in Italian of the task was administered online via a secure platform (i.e. Limesurvey), ensuring anonymity and confidentiality of participant data. Responses were collected and coded for statistical analysis.

**Data Analysis**

Internal consistency was assessed by calculating Cronbach's alpha for the responses to the three items. Descriptive statistics for age were computed, and the gender distribution was analyzed. A chi-square test was employed to evaluate any significant differences in gender distribution.

**Results**

The Italian version of the questionnaire demonstrated acceptable internal consistency, with a Cronbach's alpha of 0.693. This suggests that the translation did not substantially alter the psychometric properties of the original instrument. Participants' demographic characteristics are reported in Table 1.

**Discussion**

The findings indicate that the Italian version of the "Intertemporal Decision Making Task" maintains a level of internal consistency comparable to the original English version reported by Boyle et al. [10]. The Cronbach's alpha of 0.693 is acceptable for a questionnaire with a limited number of items, suggesting that the instrument reliably measures the construct of intertemporal decision-making in an Italian context.

The diverse age range and balanced gender distribution enhance the external validity of the instrument. However, the small sample size of 30 participants represents a limitation, and the results should be interpreted with caution. The lack of significant gender differences implies that the instrument performs consistently across male and female participants in this sample.

**Conclusions**

This pilot study provides preliminary evidence supporting the reliability and validity of the Italian version of the Intertemporal Decision Making Task. The acceptable internal consistency and the balanced demographic characteristics suggest that the instrument is suitable for use in Italian populations. Further research with larger samples is recommended to confirm these findings and to explore the instrument's construct and criterion validity in more depth.

**References**

1. Boyle, P. A., Yu, L., Gamble, K. J., & Bennett, D. A. (2013). Temporal discounting is associated with an increased risk of mortality among community-based older persons without dementia. *PLoS One*, *8*(6), e67376.
